# Estimated costs of advanced lung cancer care in a public reference hospital

**DOI:** 10.1590/S1518-8787.2017051006665

**Published:** 2017-08-03

**Authors:** Renata Erthal Knust, Margareth Crisóstomo Portela, Claudia Cristina de Aguiar Pereira, Guilherme Bastos Fortes

**Affiliations:** I Coordenação de Assistência. Instituto Nacional de Câncer José Alencar Gomes da Silva. Rio de Janeiro. RJ, Brasil; II Departamento de Políticas, Planejamento, Gestão e Práticas em Saúde. Escola Nacional de Saúde Pública Sérgio Arouca. Fundação Oswaldo Cruz. Rio de Janeiro. RJ, Brasil; III Centro de Pesquisa Clínica. Instituto Nacional de Câncer José Alencar Gomes da Silva. Rio de Janeiro. RJ, Brasil

**Keywords:** Lung Neoplasms, economics, Carcinoma, Non-Small Cell Lung, economics, Health Care Costs, Unified Health System

## Abstract

**OBJECTIVE:**

To estimate the direct medical costs of advanced non-small cell lung cancer care.

**METHODS:**

We assessed a cohort of 277 patients treated in the Brazilian National Cancer Institute in 2011. The costs were estimated from the perspective of the hospital as a service provider of reference for the Brazilian Unified Health System. The materials and procedures used were identified and quantified, per patient, and we assigned to them monetary values, consolidated in phases of the assistance defined. The analyses had a descriptive character with costs in Real (R$).

**RESULTS:**

Overall, the cohort represented a cost of R$2,473,559.91, being 71.5% related to outpatient care and 28.5% to hospitalizations. In the outpatient care, costs with radiotherapy (34%) and chemotherapy (22%) predominated. The results pointed to lower costs in the initial phase of treatment (7.2%) and very high costs in the maintenance phase (61.6%). Finally, we identified statistically significant differences of average cost by age groups, education levels, physical performance, and histological type.

**CONCLUSIONS:**

This study provides a current, useful, and relevant picture of the costs of patients with non-small cell lung cancer treated in a public hospital of reference and it provides information on the magnitude of the problem of cancer in the context of public health. The results confirm the importance of radiation treatment and hospitalizations as the main components of the cost of treatment. Despite some losses of follow-up, we assess that, for approximately 80% of the patients included in the study, the estimates presented herein are satisfactory for the care of the disease, from the perspective of a service provider of reference of the Brazilian Unified Health System, as it provides elements for the management of the service, as well as for studies that result in more rational forms of resource allocation.

## RESUMO

**OBJETIVO:**

Estimar os custos médicos diretos da assistência ao câncer de pulmão não pequenas células avançado.

**MÉTODOS:**

Foi avaliada uma coorte de 277 pacientes matriculados no Instituto Nacional do Câncer em 2011. Os custos foram estimados sob a perspectiva do hospital como prestador de serviços de referência para o SUS. Insumos e procedimentos utilizados foram identificados e quantificados, por paciente, sendo a eles atribuídos valores monetários, consolidados por fases da assistência definidas. As análises tiveram caráter descritivo com custos em reais (R$).

**RESULTADOS:**

Em termos globais, a coorte representou um custo de R$2.473.559,91, sendo 71,5% relacionados à atenção ambulatorial e 28,5% as internações. Na atenção ambulatorial, predominaram os custos com radioterapia (34%) e quimioterapia (22%). Os resultados apontaram para custos menores na fase inicial de tratamento (7,2%) e custos muito elevados na fase de manutenção (61,6%). Por fim, identificaram-se diferenças estatisticamente significativas das médias dos custos por faixas etárias, níveis de escolaridade, desempenho físico e tipo histológico.

**CONCLUSÕES:**

Este estudo fornece um retrato atual, útil e relevante sobre os custos de pacientes com câncer de pulmão não pequenas células assistidos em um hospital público de referência e provê elementos sobre a magnitude do problema do câncer no âmbito da saúde pública. Os resultados ratificam a importância do tratamento radioterápico e das internações como principais componentes de custo do tratamento. Apesar de algumas perdas de seguimento avalia-se que, para cerca de 80% dos pacientes incluídos no estudo, as estimativas aqui apresentadas sejam satisfatoriamente fidedignas ao cuidado da doença, sob a perspectiva de um prestador de referência do SUS, fornecendo elementos para a gestão do serviço, bem como para estudos que redundem em formas mais racionais de alocação de recursos.

## INTRODUCTION

Cancer is an important public health problem in the world, and it is estimated the occurrence of 27 million of incident cases and 12,600,000 deaths from the disease globally for 2030, among which 2,400,000 (19.0%) will be from trachea, bronchus, and lung cancer^20^. In Brazil, it is estimated the occurrence of approximately 600,000 new cases of cancer for the biennium 2016-2017, being 28,220 cases of lung cancer, of which 17,330 in men and 10,890 in women^8^.

This work focuses on lung cancer, which is the most common of all malignant tumors, showing an increase of 2% per year in its worldwide incidence^8^. According to data from the Instituto Nacional de Câncer José Alencar Gomes da Silva (INCA – National Cancer Institute José Alencar Gomes da Silva), lung cancer became one of the leading causes of avoidable death at the end of the 20th century, since it is associated with smoking. The diagnosis is often in advanced stages, since the symptoms in the early stages of the disease are not common, increasing the likelihood of debilitating symptoms, failure of interventions, and unfavorable outcomes. Because of the diagnosis in advanced stages, most of the patients are not candidates for curative treatment, requiring palliative treatment with radiotherapy and chemotherapy, with a fundamental role in increasing overall survival. The disease remains highly lethal, with a mortality rate of approximately 90%^8^. Finally, it is worth highlighting that patients with non-small cell lung cancer (NSCLC) represent approximately 85% of all patients with lung cancer^11,13^.

Tobacco use is the main risk factor for cancer, being related, in the world, to more than 20% of cancer deaths and 71% of lung cancer deaths. Its consumption is responsible for 90% of cases of lung cancer in men and 79% of cases in women; the risk for secondhand smoke is estimated as 30% higher compared to that for individuals not exposed to tobacco^1,5^.

In addition to the loss of life caused by the deaths from cancer, associated with economic losses that are difficult to measure, the financial cost of the disease is substantial and is a great challenge, especially for health systems with universal access, as in the case of Brazil. The care of the cancer patient incurs many expenses to meet the high disease burden, which must be faced in a scenario of growing investment needs, finite resources, and the search for more effective and efficient strategies.

The aim of this study was to estimate the direct costs of the care for advanced NSCLC (stage IIIB/IV) from the perspective of a public hospital of reference for the Brazilian Unified Health System (SUS), considering the initial (diagnosis), treatment maintenance, and terminal phases, and to explore the relations between costs and demographic and clinical variables.

## METHODS

The study was based on secondary data of a retrospective cohort of patients diagnosed with NSCLC stage IIIB/IV who were treated at the Thorax Section of the INCA between January 1 and December 31, 2011. The estimates were carried out from the survey of the cost per patient, from the perspective of the INCA – Hospital do Cancer I^a^ – as a service provider of reference for the SUS.

We used a bottom-up approach, in which the cost is based on individual units, which were collected directly from the patient sample. This approach estimates the costs by calculating the average cost. Thus, we multiply the unit cost of a treatment (resource) by the amount of use of the resource to obtain an estimate of the average cost of the treatment.

As the analysis perspective was the hospital as a service provider, only direct medical costs were included in the analysis, including: drugs, appointments, administration of infusion protocols, services and procedures provided, laboratory, imaging tests, blood transfusion, and hospitalization.

We did not include information about direct non-medical costs (transportation and food costs) or related to other perspectives of analysis, such as out-of-pocket costs of patients or family members and indirect costs, such as loss of productivity because of the inability to work resulting from treatment or loss of economic productivity related to premature death.

Of the 484 patients confirmed with NSCLC, there was a 2.5% loss (n = 12) from missing medical records. In this way, the potentially eligible population for the study comprised 472 patients, who were then submitted to the following inclusion criteria: patients aged over 18 years, diagnosis of stage IIIB/IV reported in the medical record, patients with physical performance from zero to two, and patients who started treatment or follow-up at INCA after diagnosis. Specifically, the scale of performance status assessment of the patient is used to assess how the disease is progressing and affecting the daily life skills of the patient, ranging from zero (fully active patient) to five (death)^14^. Patients participating in clinical trials and patients diagnosed with second primary tumor, regardless of the year of diagnosis, were excluded from the study.

According to Drummond et al.^6^, cost estimate involves three stages: the identification of categories of resources relevant to the assessment; the measurement of the amounts of resources used, in physical units; and, the valuation of the resources consumed in monetary terms. The costs are estimated by multiplying the amount of units consumed by unit price^6,10^. In this way, the resources used were identified and quantified according to their consumption in the three care phases established for this study: initial (diagnosis), maintenance (treatment), and terminal (three months prior to death), and we assigned monetary values to them.

The study comprised 18 months of observation, from the first appointment of the patient at INCA. The end of follow-up may have been motivated by death, referral to palliative care unit, or last assessment recorded, either by loss of follow-up or the end of the observation period defined.

The data were collected from medical records and information systems available, such as the material management system of INCA, the Enterprise Management System (EMS), from TOTVS, the consumption management system, the Business Intelligence (BI) from IBM, and the administration management system, the Absolut Interconnected from Alert. Table 1 systematizes the cost items, how these items were valued, their sources of data, and the degree of uncertainty about the estimates.

**Table 1:** Valuation of the procedures, services, and materials consumed; sources of data; and, degrees of uncertainty in the estimates.

Item	Valuation^a^	Source(s)	Degree of uncertainty^b^
Drugs	Average unit price/Dose	System of BI	Low
Laboratory tests^c^	Contract for lease and maintenance of equipment/unit price and contract for third-party tests (except for serological tests) We also considered the cost of direct labor per test	Absolut and Intranet system, module of clinical applications – clinical pathology	Low (hematological/biochemical) Average (serological/immunochemical)
Pathological anatomy^d^	Unified table of strategic procedures, drugs, and materials of the Unified Health System	Absolut and Intranet system, module of clinical applications – clinical pathology	High
Imaging tests^e^ (except positron emission tomography/PETCT)^f^	Unified table of strategic procedures, drugs, and materials of the Unified Health System	Medical record, Absolut and Intranet system, module of clinical applications – imaging tests	High
Appointments^g^ (all specialties and professional profiles)	Outpatient: cost of appointment/hour Emergency: cost of service/hour Hospitalization: cost of outpatient service is assumed	Medical record and Absolut and Intranet system – direct research to the record	Average
Other tests and procedures^h^ (transfusion of blood/blood products)	Unified table of strategic procedures, drugs, and materials of the Unified Health System	Medical record, Absolut and Intranet system – direct research to the record	High
Radiotherapy	Planning: secondary radiotherapy procedures of the unified table of strategic procedures, drugs, and materials of the Unified Health System Treatment: primary radiotherapy procedures of the unified table of strategic procedures, drugs, and materials of the Unified Health System Cost/session Cost/radiated field	Medical record, Absolut and Intranet system – direct research to the record and authorization of individual outpatient procedures	Average
Chemotherapy	Cost of administration	System of BI, Enterprise Management System Medical record, Absolut and Intranet system – direct research to the record	Low

^a^ Values for 2014.

^b^ The assessment criteria for the degree of uncertainty were subjective from the source of data available. The data with a low degree of uncertainty were those in which the average unit cost of resources used was properly collected with updated data from information systems. The data with high degree of uncertainty had as reference the Unified Table of Strategic Procedures, Drugs, and Materials of the Unified Health System, for cases in which the cost could not be obtained. The average degree of uncertainty was considered in cases in which multiple sources were used for valuation.

^c^ The laboratory tests, including hematological, biochemical, serological, immunological, and urine tests, amounted to 15,373 in outpatient and 4,352 in hospitalization, being 19,725 tests in total.

^d^ Approximately 735 pathological tests and procedures were carried out for outpatient service and 15 for hospitalization, amounting to 750 tests and procedures.

^e^ Considering the three phases included, 1,328 imaging tests were carried out at the outpatient level and 300 at the hospitalization level, amounting to 1,628 imaging tests.

^f^ Unit cost of the procedure according to the salary career perspective of Science and Technology (R$3,150.30)^3^.

^g^ Approximately 3,485 outpatient and 3,311 hospitalization appointments were carried out, amounting to 6,796 appointments. Of the set of appointments, most occurred during hospitalization in the terminal phase – 2,620 appointments.

^h^ Approximately 64 blood transfusion procedures were carried out in outpatient and 57 in hospitalization, amounting to 121 procedures.

In addition to the items listed in the Table, we computed, with a low degree of uncertainty, the daily hospital costs, from the data of the Brazilian Federation of Hospitals. We considered the daily basic ward type, including rooms with standard furniture, bed and bath linen for the patient, concurrent and terminal sanitization, oral diet of the patient, and nursing care.

Additionally, this study considered that some materials were consumed with fixed coefficients, addressed as kits. The value of each kit was obtained and added to the final cost of the procedure related, for a closer approximation of the actual cost. The components of the kits were established from consulting the specialists responsible for the related procedures, in addition to consulting the Standard Operating Procedures and Regulatory Service Instructions, available in the Sistema Normatizado do Serviço de Auditoria Interna e Gestão da Qualidade (Normative System of the Internal Audit and Quality Management Service), on the Intranet of the INCA. We considered that each patient used materials without sharing.

At the end of the inclusion of these data, they were extracted in various customized tables in Microsoft Access 2013^®^, in which calculations were automated. The tables were then converted into relational databases for analysis in the Statistical Analysis System (SAS^®^), version 9.3b.

The analyses presented herein have a descriptive nature, focusing on the distributions of socio-demographic and clinical variables and, centrally, on the costs (expressed in Real) of the disease care in the study population. For the categorized variables, we obtained the frequencies and corresponding percentages, and, for the continuous variables, the ranges of observed values, averages, standard deviations, and quartiles.

To determine differences in the care costs for NSCLC by the socio-demographic and clinical variables, we used the Kruskal-Wallis test to compare the means. We considered the significance level α = 0.05, with statistically significant differences denoting the rejection of the hypothesis of equality between at least two groups.

The study was designed in accordance with the guidelines and standards governing research involving human beings (Resolution CNS 466/12) and initially submitted to the Research Ethics Committee of the Fundação Oswaldo Cruz (record CAAE 31042514.3.0000) and, subsequently, to the Research Ethics Committee of the INCA (record CAAE 524031042514.3.3001.5274).

## RESULTS

Of the 472 patients with NSCLC initially selected, 277 (58.7%) were eligible for this study after the inclusion and exclusion criteria proposed.

We observed that 63.5% of the patients were aged between 50 and 69 years. The minimum and maximum age recorded were 36 and 86 years, respectively, being the average 62.8 years (SD = 9.9) and the median 63 years. Males were predominant, accounting for 61.4% of the cases. In relation to race, 65.7% of the patients were white, followed by 28.5% brown. As for education level, almost half of patients analyzed (49.8%) had only incomplete elementary school, followed by complete elementary school (26%), complete high school (11.9%), and higher education (6.5%). Illiterate patients were 5.8% of the population.

Among the patients who claimed to have used tobacco throughout their lives (90% of the population), more than 50% still had this habit in the screening. As for smoking time, the average was 39.8 years of use and the median was 40 years, ranging between five and seventy years. The average number of packs smoked per year was 53.9, and the median was 48, ranging from half a pack/year and 180 packs/year. In relation to the stage of the disease, 32.9% had stage IIIB, and 67.1% had stage IV. Regarding histology, a little more than half (58.9%) had the adenocarcinoma subtype, followed by squamous cell carcinoma (35%). Only 4.3% of the patients presented the large-cell carcinoma subtype. In 1.8% of cases classification was not possible, as the subtype was not specified in the histopathological report. In the screening, 133 (48.0%) patients had some type of comorbidity, being cardiovascular diseases the most frequent (84.97%), followed by metabolic diseases (21.80%), especially diabetes mellitus.

**Figure 1:**
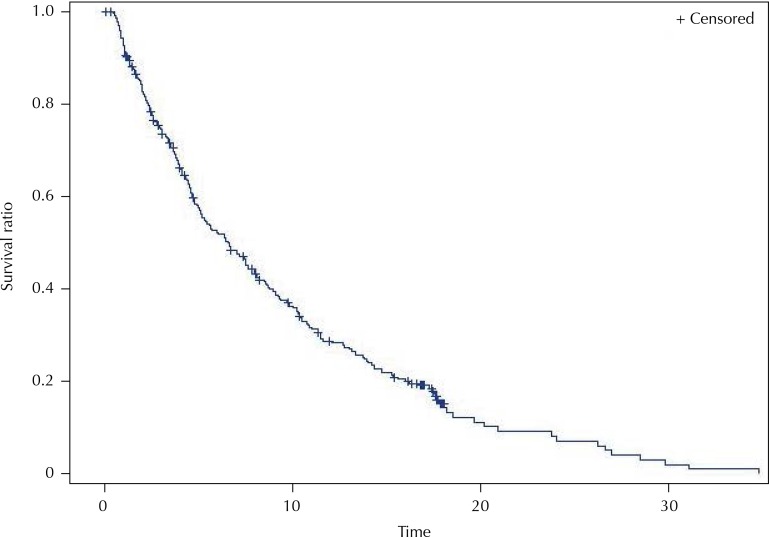
Survival curve (in months) of patients with advanced non-small cell lung cancer treated in the National Cancer Institute José Alencar Gomes da Silva (INCA) based on the Kaplan-Meier method.

In the 18 months of observation of the cohort, 77.6% of patients died: 25% of patients died in up to 2.9 months 50%, up to 6.6 months, and 75% up to 13.8 months, showing a relatively very high short-term lethality (Figure 1).

Total cost was R$2,473,559.91, being R$1,769,526.22 (71.5%) related to outpatient care and R$704,035.69 (28.5%) to hospital care (hospitalization) (Table 2). Regarding distribution by treatment phases, 7.2% of the amount was used in the initial phase of treatment, 61.6% in the maintenance phase, and 31.2% in the terminal phase. Outpatient care make up most of the cost of the initial phase (66.5%) and maintenance phase (90.2%), while hospitalizations represent the largest share (64.2%) of the total cost associated with the terminal phase.

**Table 2:** Distribution of total outpatient and hospitalization costs. (N = 277)

Phase	Total outpatient and hospitalization costs.					

	Average (R$)	SD (R$)	Q1 (R$)	Median (R$)	Q3 (R$)	Sum (R$)
Initial						
Outpatient	426.37	497.26	24.00	359.87	638.43	118,104.18
Hospitalization	214.61	877.54	0	0	0	59,447.96
Total	640.98	1,100.73	24.00	379.95	678.17	177,552.14
Maintenance						
Outpatient	4,962.69	13,488.59	0	1,180.83	4,448.76	1,374,664.37
Hospitalization	538.60	1,776.57	0	0	0	149,193.38
Total	5,501.29	13,830.13	0	1,274.42	6,152.97	1,523,857.75
Terminal						
Outpatient	999.12	1,588.13	0	396.31	1,485.67	276,757.67
Hospitalization	1,788.42	2,826.82	0	0	3,116.92	495,392.35
Total	2,787.55	3,597.06	0	1,219.04	4,896.02	772,150.02

Total	8,929.82	13,660.59	2,459.31	5,887.78	9,823.14	2,473,559.91

SD: standard deviation; Q1: first quartile; Q3: third quartile

The cost of treating a patient with advanced NSCLC at INCA ranged from R$101.71 to R$90,861.72, being the average R$8,929.82 and the median R$5,887.78. A significant part of the average individual cost is related to the outpatient care of the maintenance phase, in which the costs with chemotherapy and radiotherapy are concentrated.

In relation to the cost of outpatient care, the components that contributed the most were radiotherapy (34%) and chemotherapy (22%), followed by drugs (12%), imaging tests (11%), laboratory tests (10%), pathological examinations (5%), outpatient consultations (4%), other tests or procedures (2%), and blood transfusion (0.1%).

Despite the survey of costs being restricted to the period of 18 months after screening the patient, death dates were obtained beyond that period. This allowed the classification of patients into groups based on the time of death and time of observation, in the study. For 78% of the patients – those who died within 18 months and those who died after 18 months after screening, but who had a record of referral to the palliative care unit – we assume that the cost estimates obtained reflect the full treatment of the patients. For the remaining 22% (last four groups), we highlight the possibility of underestimation of costs.

**Figure 2:**
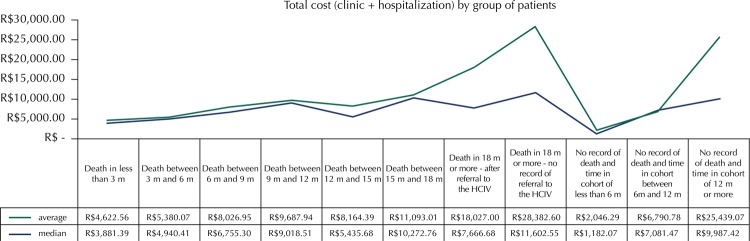
Average and median total costs per group of patients. (N = 277)

Figure 2 shows that the average total cost of oncologic care ranged from R$4,622.56, in cases in which death occurred within three months after the admission of the patient, to R$18,027.00, in cases in which death occurred after 18 months, but with record of referral to the HCIV (palliative care unit). These estimates reflect the actual costs of the patient care, being considered reliable for the treatment of the disease.

HCIV: Hospital do Cancer IV

Time in m = months

Table 3 shows the comparison of the averages of the total cost of treatment by categories related to the socio-demographic, behavioral, and clinical variables, based on the Kruskal-Wallis test.

**Table 3:** Comparison of the averages of the total cost using the Kruskal-Wallis test. (N = 277)

Variable	n	Average (R$)	SD (R$)	Median (R$)	Kruskal-Wallis test (p)
Gender					
Female	107	9,419.55	15,251.62	5,474.62	0.6651
Male	170	8,621.58	12,594.40	6,049.55	
Age					
< 50	33	9,061.57	11,985.83	7,622.05	0.0280
50–59	73	9,009.86	10,972.60	7,076.66	
60–69	103	10,088.24	16,170.49	6,198.43	
70–79	55	6,638.39	12,409.48	3,753.86	
≥ 80	13	8,662.20	15,337.53	3,306.82	
Education level					
Illiterate	16	9,063.51	22,091.49	3,027.17	0.0430
Incomplete elementary school	138	8,773.83	14,248.46	5,014.75	
Complete elementary school	72	7,994.75	9,912.50	6,818.30	
Complete high school	33	10,308.76	14,636.36	7,339.87	
Complete higher education	18	11,219.12	11,671.07	8,843.79	
Race					
Brown	79	10,260.81	16,581.59	6,198.43	0.6314
White	182	8,659.06	12,766.09	5,453.35	
Black	16	5,437.89	3,761.72	6,424.66	
Marital status					
Married	177	8,567.07	12,807.04	5,900.67	0.7349
Divorced or separated	22	12,811.26	20,602.64	5,983.84	
Widow	33	9,621.57	16,049.02	7,266.68	
Single	45	7,951.75	10,731.79	5,345.12	
Stage					
IIIB	91	7,691.67	5,565.19	7,341.49	0.0908
IV	186	9,535.58	16,193.03	5,238.31	
Physical performance					
0	39	9,523.37	7,185.47	8,066.68	0.0002
1	156	10,756.39	17,268.31	6,433.75	
2	39	5,065.40	4,408.21	3,778.48	
Histological type					
Adenocarcinoma	163	10,871.40	17,158.89	6,354.98	0.0574
Squamous cell carcinoma	97	6,186.98	4,281.73	5,432.09	
Large-cell carcinoma	12	3,864.94	3,337.29	2,933.85	
Ignored	5	11,001.20	7,929.51	7,718.49	
Smoking habit					
Smoker	145	7,555.87	9,393.62	5,474.62	0.6889
Former smoker	104	7,270.58	7,845.40	6,433.75	
Non-smoker	28	22,207.83	31,600.49	6,203.32	
Number of comorbidities					
0	144	8,777.44	13,078.25	5,932.71	0.7742
1	108	8,455.50	12,692.77	5,453.35	
≥ 2	25	11,974.23	19,980.02	6,885.80	
Palliative care referral					
No	155	10,718.30	16,887.24	6,861.31	0.0149
Yes	122	6,657.57	7,307.63	4,619.02	

It is possible that in the case of older patients, the lower costs are related to faster death. In the case of education level, patients with higher education were associated with the highest average and median costs, perhaps reflecting greater ability to demand treatment resources. The results show a clear gradient between the medians of the variable, indicating predominantly lower costs for illiterate patients, which grows with educational level. It is possible that patients with lower education have more difficulty in accessing the service and become more debilitated. Regarding the situation of smoking (smoker and non-smoker) and the number of comorbidities, no significant differences were found between the costs of different patients.

Stage showed a borderline association (significant at α = 0.10 and not significant at α = 0.05) with cost of treatment, showing a trend of higher average cost among patients with stage IV than with stage IIIB; although, when we consider the median, the situation is reverse, perhaps reflecting faster death in the most advanced stage of the disease.

The comparison of average costs between the histological subtypes points to a higher value for the treatment of adenocarcinoma, while the relative comparison of the level of physical performance in the screening indicates lower cost among more debilitated patients (physical performance = 2), probably because of faster death.

Furthermore, we considered the possibility of differentiation of the cost of treatment between patients with and without record of referral to the palliative care hospital unit, with lower cost for the first ones, denoting again the selection of patients who tended to die more quickly.

As indicated in Table 1, the items of costs used in this study have different degrees of uncertainty on the estimates found. More inaccurate items need to be better systematized or must be the central focus of sensitivity analyses in the use of estimates.

## DISCUSSION

This study systematized the use of resources and associated costs, obtained in the follow-up, for up to 18 months, of a cohort of 277 patients with advanced NSCLC, treated at INCA in 2011. There was loss of follow-up of some patients included in the study, while others were still alive after the observation period established. However, nevertheless, we assess that, for approximately 80% of the patients, the estimates presented herein are satisfactory for the care of the disease, from the perspective of a service provider of reference for the SUS.

This analysis provides a current, useful, and relevant picture of the costs with care for patients with NSCLC treated in a public hospital of reference and it provides information on the magnitude of the problem of cancer in the context of public health. We hope to make a significant contribution to future studies involving costs or cost-effectiveness of interventions for lung cancer.

In relation to the characteristics of the population, we can infer that they are comparable with other national studies in relation to the distribution by gender – with male predominance –, age, stage in the initial screening, histological types, time of smoking, and smoking burden, in addition to physical performance at diagnosis^11,15,b,c^.

Regarding the comparison analyses of cost averages by socio-demographic and clinical variables, statistically significant differences were identified only for age, education level, histological type, and physical performance at diagnosis.

The medians of the costs presented negative association with age, being higher the younger the age groups. The cost averages do not have a linear behavior, but they are lower among patients aged 70 years or more, probably reflecting a shorter survival.

The difference in the cost averages by education level was an interesting and intriguing finding of this study. According to the results found, the higher the education level of the patient, the greater were the costs. It is possible that patients of low educational level had more difficulty to access the service or were more debilitated. It is also possible that patients with high education level had less difficulty entering the service and actively demanded more care. This is a finding that speaks against equity and that deserves to be better explored and understood.

The findings presented in this study are not easily comparable with those of other national and international studies, whether because of methodological differences or because of the diversity of contexts. They ratify the importance of radiation treatment and hospitalizations as the main components of the cost of treatment of patients with lung cancer, according to a previous national study^b^, although estimated costs and percentages of participation of different components can vary from the profile of the disease considered. In general terms, the main methodological differences between studies that focus on the costs of treatment of lung cancer are pertinent to the profile of the disease, the selection of patients, the time horizon and perspective of the analysis, and the cost items considered, as well as the potential clinical predictors selected^2,7,9,12,16-21^.

Generally speaking, we can see from the literature that costs with cancer treatment decrease significantly with the stage and extension of the tumor, being greater in the first year after diagnosis and in the final phase of the disease^4^. This trend of the costs suggests that health services must be prepared to bear the initial costs of patients, which extend up to the first year after diagnosis. After this period, the costs tend to stabilize, even in active treatment. Another trend is that these costs increase in the terminal phase, especially because of repeated hospitalizations.

Regarding the survey of data, some limitations related to retrospective collection were confirmed, such as the loss of information about resources consumed by patients because of incomplete medical records. On the other hand, the use of retrospective data had the advantage of avoiding influences and representing the actual clinical practice.

Another important limiting factor was the lack of an integrated system of information that could allow the integration of various cost centers and provide more accurate data on the materials and costs involved in the procedures. The data were taken from different sources, with possible loss of information. In addition, because of the lack of some data that could be changed into costs, we used information from the unified table of the SUS to estimate the value of some procedures.

The selected hospital is specialized and of reference in high complexity oncology care within the SUS, which limits the generalization of results, since each hospital registered for oncology care in the Ministry of Health is free to incorporate clinical treatment guidelines and, thus, offer therapeutic schemes that are different from other units. However, the costs estimated herein can reflect the profile of the costs in public health.

It is important to note that costs should not be the main focus of discussion, and they should not be used in isolation in order to change clinical recommendations for the diagnosis and treatment of cancer, but they must help in the search for strategies that re-evaluate public health policies and stimulate the price negotiation policy and remuneration of the unified table. The identification of a high volume of expenses does not provide, in itself, enough information to suggest good use of the resource. The results about costs are not meant to show that spending on a certain area corresponds to an efficient application of resources, serving only as a descriptive base. Therefore, it is critical the concern with the prioritization and allocation of resources, taking into account the reality of scarcity to meet health needs.

## Footnotes

a The hospital units of the INCA are part of the SUS and offer full treatment to persons who have cancer. They are located in the city of Rio de Janeiro, Brazil, and are classified as of high complexity. The Hospital do Câncer I treats children with various types of cancer and adults with cancer in regions of the digestive system, head and neck, chest, and skin, in addition to providing service in oncologic neurosurgery, oncologic urology, oncologic hematology, radiotherapy, and brachytherapy. Address: Praça Cruz Vermelha 23 - Centro. Rio de Janeiro, Brazil.

b Pinto MFT. Custos de doenças tabaco-relacionadas: uma análise sob a perspectiva da economia e da epidemiologia [these]. Rio de Janeiro: Escola Nacional de Saúde Pública Sergio Arouca; 2007 [cited 2017 Mar 10]. Available from: http://www.arca.fiocruz.br/handle/icict/4354

c Mora PAR. Análise de sobrevida de pacientes com câncer de pulmão [dissertation]. Rio de Janeiro: Universidade Federal do Rio de Janeiro; 2004 [cited 2017 Mar 10]. Available from: http://bvsms.saude.gov.br/bvs/publicacoes/inca/Paulo_Alexandre_Ribeiro_Mora.pdf
